# Medical residency in Portugal: a cross-sectional study on the working conditions

**DOI:** 10.3389/frhs.2023.1190357

**Published:** 2023-12-05

**Authors:** José Chen-Xu, Bruno Miranda Castilho, Bruno Moura Fernandes, Diana Silva Gonçalves, André Ferreira, Ana Catarina Gonçalves, Maycoll Ferreira Vieira, Andreia M. Silva, Fábio Borges, Mónica Paes Mamede

**Affiliations:** ^1^ Unidade de Saúde Pública, Agrupamento de Centros de Saúde Baixo Mondego, Coimbra, Portugal; ^2^ Comprehensive Health Research Centre, National School of Public Health, NOVA University of Lisbon, Lisbon, Portugal; ^3^ Cardiology Department, Hospital Distrital de Santarém, Santarém, Portugal; ^4^ Radiology Oncology Department, Centro Hospitalar Universitário de Coimbra, Coimbra, Portugal; ^5^ Unidade de Saúde Familiar ARS Médica, Agrupamento de Centros de Saúde Loures-Odivelas, Loures, Portugal; ^6^ Medical Oncology Department, Centro Hospitalar de Lisboa Ocidental, Lisboa, Portugal; ^7^ Infectious Diseases Department, Centro Hospitalar Universitário de Lisboa Central, Lisboa, Portugal; ^8^ Centros de Saúde de Santana e do Caniçal, Serviço de Saúde da Região Autónoma da Madeira, EPERAM, Madeira, Portugal; ^9^ General Surgery Department, Hospital da Horta, EPER, Açores, Portugal; ^10^ Unidade de Saúde Familiar S. Miguel-O-Anjo, Agrupamento de Centros de Saúde Ave-Famalicão, Famalicão, Portugal; ^11^ Anaesthesiology Department, Centro Hospitalar de Lisboa Central, Lisboa, Portugal

**Keywords:** medical education, residency, training, health policy, health economics, healthcare workers

## Abstract

**Objectives:**

The current European crisis in human resources in health has opened the debate about working conditions and fair wages. This is the case with Resident doctors, which have faced challenges throughout Europe. In Portugal, they account for about a third of the doctors in the Portuguese National Health Service. No studies to date objectively demonstrate the working conditions and responsibilities undertaken. This study aims to quantify the residents' workload and working conditions.

**Methods:**

Observational, retrospective cross-sectional study which involved a survey on the clinical and training activity of Portuguese residents, actively working in September 2020. The survey was distributed through e-mail to residents' representatives and directly to those affiliated with the Independent Union of Portuguese Doctors. The descriptive analysis assessed current workload, and logistic regression models analyzed associations with geographical location and residency seniority.

**Results:**

There were a total of 2,012 participants (19.6% of invited residents). Of the residents giving consultations, 85.3% do so with full autonomy. In the emergency department, 32.1% of the residents work 24 h shifts and 25.1% work shifts without a specialist doctor present. Regarding medical training, 40.8% invest over EUR 1,500 annually. Autonomy in consultations was associated with being a Family Medicine resident (OR 4.219, *p *< 0.001), being a senior resident (OR 5.143, *p *< 0.001), and working in the Center (OR 1.685, *p *= 0.009) and South regions (OR 2.172, *p *< 0.001). Seniority was also associated with investing over EUR 1,500 in training annually (OR 1.235, *p *= 0.021).

**Conclusion:**

Residents work far more than the contracted 40 h week, often on an unpaid basis. They present a high degree of autonomy in their practice, make a very significant personal and financial investment in medical training, with almost no time dedicated to studying during working hours. There is a need to provide better working conditions for health professionals, including residents, for the sake of the sustainability of health systems across Europe.

## Highlights

• Senior residents present higher autonomy and perform more 24 h emergency shifts.
• Seniority in residency was associated with higher expenditure in training.
• Regional disparities in residency were present in ER shifts and extra hours.
• Working conditions must be improved to avoid brain drain of doctors across Europe.

## Introduction

Medical residency in Portugal begins after completing the six-year long degree in medicine and one year of general training internship. It is a post-graduate medical training internship that lasts between four and six years, depending on the specialty. Its purpose is to qualify physicians for technically differentiated practice in a medical specialty, and those who complete this training become medical specialists (1, 2). Due to the training of highly differentiated tasks and responsibilities, medical residency programs present their own legal framework, as the work conducted by residents needs to be tutored according to our legislation, being necessary to have a specialist doctor physically present (1).

Resident doctors (RDs) account for about 33% of the physicians in the Portuguese National Health Service (PNHS) (3). In almost all specialties, factors such as being over-burdened with work, rest deprivation and night or shift work are an integral part of medical residency (4). This leads to a clear reduction in physicians' physical and psychological well-being, which can be harmful to patients (5–7). This reality is also present in countries such as the United States (8) and the United Kingdom and all across Europe (9, 10), receiving particular attention during and in the aftermath of the COVID-19 pandemic (11).

In Portugal, there is clearly an empirical understanding of the high amount of work that RDs perform and the responsibility they undertake and of the mismatch of the income when considering the responsibilities, with a reported loss in purchasing power parity of Portuguese medical doctors when compared to other European countries (12). To date, no studies have been conducted that objectively demonstrate the work and responsibilities undertaken by RDs nor their contribution to the national health system (13).

This study, therefore, aims to characterize and quantify the work performed by RDs in the PNHS, focusing on the following aspects: degree of autonomy in care provision, paid and unpaid supplementary workload and the financial and personal investment by these physicians in their training. Furthermore, factors influencing these outcomes were also evaluated, including location of training and seniority in the residency. As such, this study will contribute to increase the little knowledge on this topic, namely related to understanding the actual workload and the working conditions of resident doctors. This can provide some insight on the needs and frailties of human resources in health, given the current crisis (12), and consequently improve the retention of doctors in public healthcare systems.

## Methods

### Study design, population and data collection

A retrospective, observational, cross-sectional study was carried out, involving the completion of a survey on clinical and training activity undertaken by Portuguese RDs up to September 2020.

The survey was distributed to RDs between 22 December 2020 and 30 April 2021 via social media and e-mailed to RD representatives and directly to RDs who were part of the Independent Union of Portuguese Doctors. Eligible doctors included residents registered in the medical council and actively working on September 2020.

The questionnaire focused on the following topics: autonomy in care provision, paid and unpaid overtime, time off and compensatory rest breaks, and personal and financial investment in medical training. We used the Portuguese physician's association definition of full autonomy: a consultation where the RD was the main stakeholder responsible for conducting the consultation and the intervention of another physician was minimal (14).

Demographics, data on the area of specialized training, and on clinical and training activity were also collected. Regions were classified according to Health Administrative regions and their representativeness, being separated in four regions: North, Center, South, and Islands, and transformed in binary variables for the inferential analysis. For residency seniority, considering that the duration of residency programs ranges from four to six years, we classified them according to their duration, making them binary: RDs were considered as junior residents in the following cases: first and second years in four-year residency programs, and first to third years in five- and six-year residency programs. RDs in the later years were considered to be senior residents.

Responses were obtained on a self-report basis, in which the respondents were asked to base their answers on their official attendance records in order to reduce subjectivity of working data. Incomplete questionnaires were automatically excluded. All data were collected after consent from the participants and in an anonymized format.

### Statistical analysis

Statistical analysis was performed using IBM® SPSS® Statistic*s*, version 28 software. For descriptive analysis, normal distribution of quantitative variables was initially assessed, with the addition of the Kolmogorov-Smirnov test for normality, and the assessment of asymmetry, flatness measures and visual histogram analysis. Given the asymmetric distribution, these variables were described using the median and interquartile range (IQR). When applicable, analyses were conducted in specific subgroups, in the case of hospital-based work for the specialties that presented work in the context, and for Family Medicine for variables specific for primary healthcare. For the description of qualitative variables, absolute and relative frequencies were used. In the inferential analysis, 95% confidence intervals (CI) were estimated for proportions, with quantitative variables being converted to binary outcomes through the definition of cut off values in cluster analysis. Bivariable and stepwise multivariable logistic regression models were then conducted to assess associations, adjusting for region and residency seniority. Results were expressed with *p*-value and Odds Ratio (OR) or Adjusted Odds Ratio (adjOR), in the cases of bivariable and multivariable regressions, respectively. A significance level was considered when *p*-value < 0.05.

### Public involvement

RDs had a vital role in spreading the survey, which encouraged the participation of the medical community during the study.

### Ethics statement

The authors declare that the procedures were followed in accordance with the updated 2013 World Medical Association Declaration of Helsinki and the STROBE guidelines, having sought approval of the Ethics Committee of Hospital da Horta. Ethical consent was given according to Helsinki Declaration in a written form. All participants were over 18 years old and provided consent in advance of participation in the study.

## Results

A total of 2,012 responses to the questionnaire were included in the study, corresponding to 19.6% of the total number of practicing RDs undergoing specialty training as of September 2020 (total of 10 275 RDs). All participants accepted the questionnaire terms and conditions.

**Figure 1:**
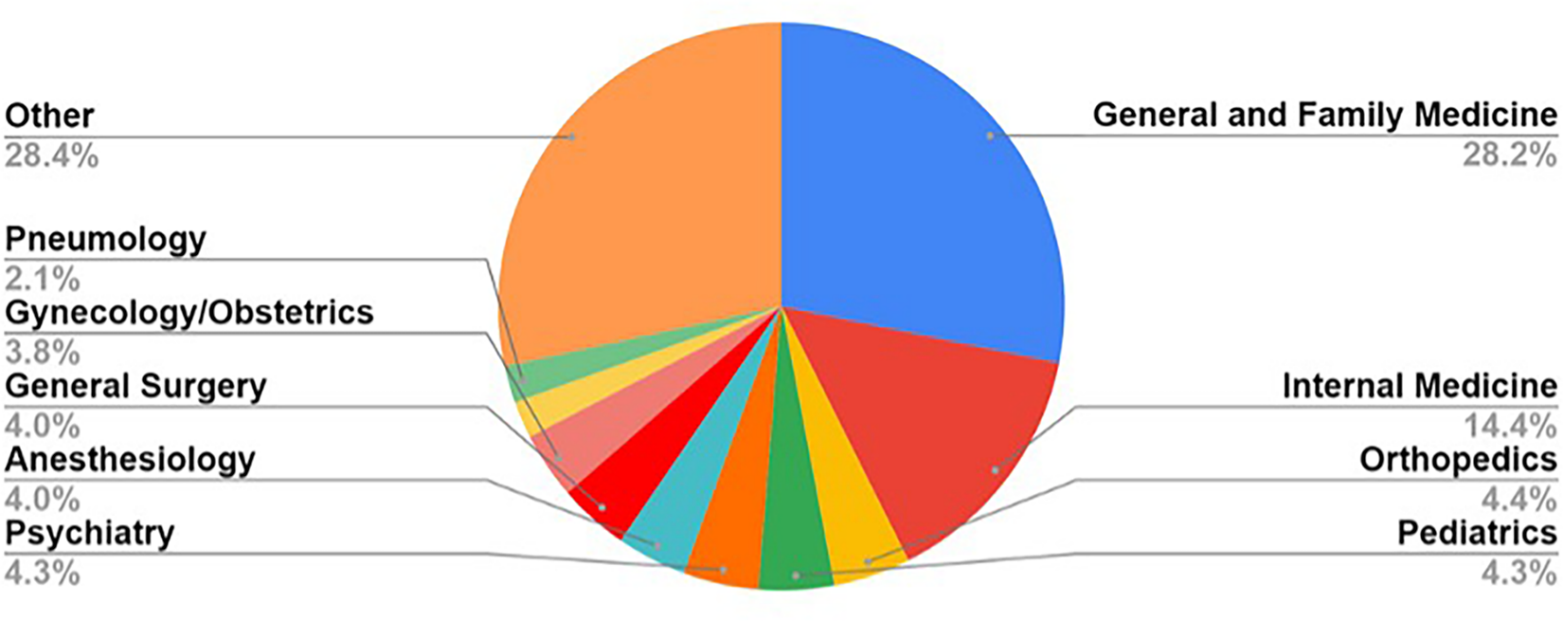
Distribution by area of specialized training within medical residency.

Figure 1 shows the distribution of participants according to the areas of specialty with the highest representation in the sample.

From the responses obtained, six RDs dropped out of the medical residency, citing reasons such as excessive workload, lack of overtime payment, excessive responsibility in early stages of training and also bullying in medical training.

Most residents belonged to the specialty of General and Family Medicine (*n *= 567, 28.2%), followed by Internal Medicine (*n *= 290, 14.4%) and Orthopedics (*n *= 88, 4.4%).

### Paid and unpaid overtime

**Figure 2:**
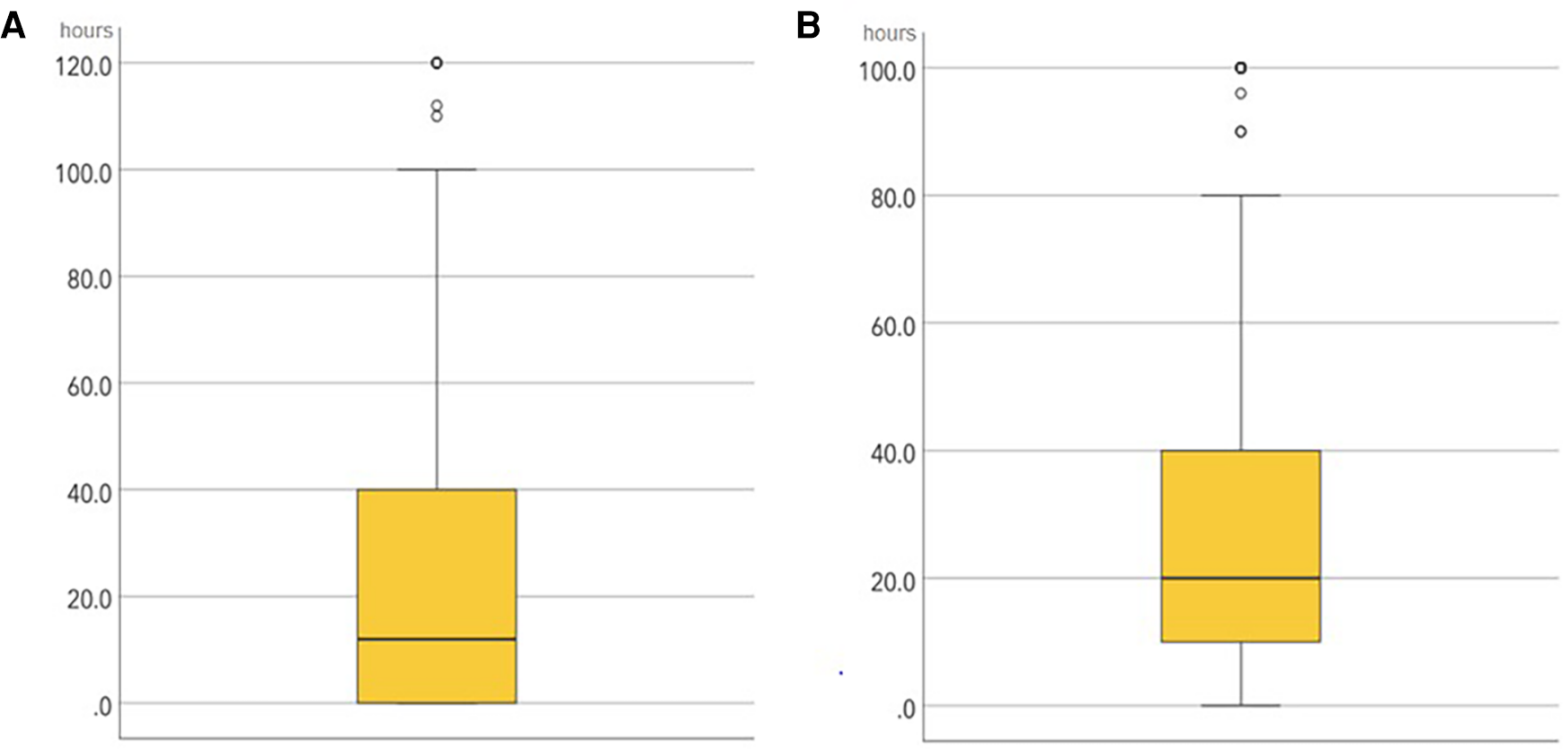
Distribution of the overtime hours per working month, showing the paid (**A**) and unpaid overtime (**B**).

From this section onwards, only the questionnaires of RDs who did not drop out of medical residency were included, which amounted to a total of 2,006 responses. Figure 2 shows the distribution of overtime hours per month, either paid (A) or not (B) to the residents.

**Graphic A showed Monthly paid overtime.** A minimum of 0 h, a maximum of 240 h, with a median of 12 h and an interquartile range (IQR) of 40 h were recorded. Four cases were excluded due to an error in the completion of this variable, assumed for a cut off value >250 h.

**Graphic B illustrates the Monthly unpaid overtime,** where a minimum of 0 h, a maximum of 240 h, with a median of 20 h and an IQR of 30 h were recorded. Four cases were excluded due to an error in the completion of this variable, assumed for a cut off value above 250 h.

### Consultations

Of the total sample, 1,767 RDs (88.1%) provided consultations in their specialty. 1,508 RDs (85.3%) performed consultations with full autonomy. The questionnaire revealed that RDs performed a median of 60 consultations with full autonomy per month, with an IQR of 90 (min: 1, max: 750).

### Hospitalization

Around 62.6% of RDs (*n *= 1,255) indicated that they were undergoing specialized training in a specialty with inpatient care. From this group, 1,015 RDs (80.9%) reported having a schedule which included infirmary work.

Table 1 summarizes the data on discharge notes and prescriptions issued in their own name, as well as invasive medical procedures done autonomously (placement of central venous catheter, collection of bone marrow aspirate, placement of temporary pacemakers, among others), on an inpatient setting.

**Table 1:** Tasks performed autonomously by resident doctors in specialty training in an inpatient setting.

Hospitalization	Number of RDs (%)	95% Confidence Interval
Autonomy to discharge	937 (93.5%)	[92.0%; 95.0%]
Outpatient prescription writing	992 (99.0%)	[98.4%; 99.6%]
In-hospital prescription writing	994 (99.2%)	[98.7%; 99.9%]
Invasive medical procedures	812 (81.0%)	[78.6%; 83.5%]

RD, resident doctor.

Most of the tasks reported above were carried out independently by the majority of residents, who performed outpatient (*n *= 992, 99.0%) and in-hospital (*n *= 994, 99.2%) prescription writing. For invasive medical procedures, 812 RDs (81.0%) stated that they had performed them autonomously. Monthly, these RDs performed a median of eight procedures, with an IQR of 18 (min: 0, max: 200).

### Emergency department

Of the completed questionnaires, 1,747 RDs (87.1%) reported working in the emergency department (ED) during their medical residency, of which 1,674 RDs (95.8%) reported having scheduled shifts. Table 2 describes the qualitative characteristics presented in the questionnaire regarding the ED, and the corresponding 95% CI.

**Table 2:** Emergency department work carried out by RDs.

ED	Number of RDs (%)	95% Confidence Interval
24 h shifts	537 (32.1%)	[29.9%; 34.3%]
Weekends	1,335 (79.7%)	[77.8%; 81.6%]
Specialist not physically present	420 (25.1%)	[23.0%; 27.2%]
Invasive medical procedures	1,266 (75.6%)	[73.6%; 77.7%]

ED, emergency department; RD, resident doctor.

For each weekend or holiday shift, there is a compensation of a day off, and for each night shift there is a compensatory rest time. The median number of untaken days off/compensatory rest per month was 1.6, with an IQR of 2 (min: 0, max: 20).

### Medical training

Resident doctors are encouraged to seek additional continuous education during the residency, as they are also evaluated for it in the final exam to become a specialist. However, many of the topics sought for are not covered in the residency, which translates into additional expenses for RDs. Table 3 describes the average annual spending on training by the 2,006 resident doctors who answered the questionnaire and who are still undergoing medical residency.

**Table 3:** Annual spending on training during residency.

Annual spending on training (EUR)	Number (Percentage)
0	2 (0.1%)
1–1,000	795 (39.6%)
1,001–1,500	391 (19.5%)
1,501–2,000	294 (14.7%)
2,001–2,500	182 (9.1%)
2,501–3,000	107 (5.3%)
>3,000	235 (11.7%)

Most RDs (*n *= 795, 39.6%) reported spending up to EUR 1,000 per year in further training, followed by EUR 1,001–1,500 (*n *= 391, 19.5%) and EUR 1,501–2,000 (*n *= 294, 14.7%). It is notable that a significant group of residents spent over EUR 3,000 on training (*n *= 235, 11.7%).

About the inclusion of study time in the work schedule during the residency, a median of 0 h per week was observed, with an IQR of 1h (min: 0h, max: 12 h).

### Geographical and seniority analysis

The geographical data and seniority of residency were utilized as independent variables to assess association with several outcomes.

Regarding paid and unpaid overtime, bivariable regression analyzes were conducted to analyze whether there were differences in the amount of hours performed, setting a cut off of 15 h (Table 4). The bivariable regression identified significant negative associations between performing over 15 unpaid extra hours per month and doing the residency in the Northern region (OR 0.64, 95% CI 0.48–0.84, *p *= 0.001) and being a senior RD (OR 0.75, 95% CI 0.58–0.97, *p *= 0.027). A positive association was found with the Southern region (OR 1.51, 95% CI 1.17–1.95, *p *= 0.001). The final multivariate regression only confirmed the association between the unpaid hours and seniority (adjOR 0.74, 95% CI 0.57–0.96, *p *= 0.025).

**Table 4:** Logistic regression model related to unpaid hours per month performed by RDs (cut off = 15).

Variable	OR	95% CI	*p*-value	adjOR	95% CI	*p*-value
Region						
North	0.64	0.48–0.84	0.001	0.74	0.52–1.06	0.102
Center	0.89	0.64–1.24	0.501			
South	1.51	1.17–1.95	0.001	1.27	0.91–1.76	0.154
Islands	1.35	0.71–2.56	0.362			
Senior RD	0.75	0.58–0.97	0.027	0.74	0.57–0.96	0.025

OR, odds ratio; adjOR, adjusted odds ratio; CI, confidence interval; RD, resident doctor.

Regarding paid hours (Supplementary Table S1), the cut off was set at 23 h, with significant associations in terms of geographical placement in the bivariable analysis: the RDs placed in the North presented a negative association (OR 0.78, 95% CI 0.64–0.94, *p *= 0.011), whereas the Southern RDs presented a higher probability of performing over 23 paid hours (OR 1.43, 95% CI 1.19–1.71, *p *< 0.001). When adjusting the model, only negative associations contributed to performing over 23 h of paid overtime work, both in the North (adjOR 0.70, 95% CI 0.57–0.85, *p *< 0.001) and in the Center of Portugal (adjOR 0.70, 95% CI 0.55–0.89, *p *= 0.003).

Considering overtime hours in the ER context, separate analyses were conducted (Supplementary Tables S2 and S3). In the case of unpaid overtime hours (cut off = 3h), the bivariable analysis found a positive association with the South region (OR 1.38, 95% CI 1.06–1.79, *p *= 0.017), which was then confirmed by the multivariable regression (adjOR 1.38, 95% CI 1.06–1.79, *p *= 0.017). For the paid hours performed in the ER by residents, a cut off of 20 h was set. The bivariable regression included positive associations with seniority (OR 1.34, 95% CI 1.08–1.67, *p *= 0.008), and being from the South (OR 1.43, 95% CI 1.15–1.78, *p *= 0.001) and Islands (OR 2.51, 95% CI 1.37–4.61, *p *= 0.003), whereas a negative association was found in the North region (OR 0.54, 95% CI 0.43–0.67, *p *< 0.001). The majority of the associations were consistent in the multivariable analysis, in regard to the North region (adjOR 0.57, 95% CI 0.45–0.72, *p *< 0.001), Islands (adjOR 2.10, 95% CI 1.14–3.88, *p *= 0.018) and seniority (adjOR 1.34, 95% CI 1.07–1.67, *p *= 0.010).

Consultations carried out with full autonomy by RDs were also analyzed (Table 5), which revealed positive associations in the bivariable analysis with being a Family Medicine RD (OR 4.42, 95% CI 2.94–6.63, *p *< 0.001), being a senior resident (OR 5.51, 95% CI 3.95–7.69, *p *< 0.001) and with regions Center (OR 1.42, 95% CI 1.01–2.01, *p *= 0.047) and South (OR 1.57, 95% CI 1.18–2.09, *p *= 0.002). There was a negative association with being a hospital RD (OR 0.23, 95% CI 0.16–0.35, *p *< 0.001) and the residency in the North region (OR 0.54, 95% CI 0.41–0.71, *p *< 0.001). When adjusting for the multivariable model, only positive associations were confirmed: being a Family Medicine RD (adjOR 4.22, 95% CI 2.78–6.410, *p *< 0.001), being a senior resident (adjOR 5.14, 95% CI 3.67–7.21, *p *< 0.001), and belonging to the Center (adjOR 1.69, 95% CI 1.14–2.49, *p *= 0.009) and South (adjOR 2.17, 95% CI 1.58–2.99, *p *< 0.001) regions.

**Table 5:** Logistic regression model related to consultations carried out autonomously by RDs.

Variable	OR	95% CI	*p*-value	adjOR	95% CI	*p*-value
Family Medicine RD	4.42	2.94–6.63	<0.001	4.22	2.78–6.41	<0.001
Hospital RD	0.23	0.16–0.35	<0.001			
Region						
North	0.54	0.41–0.71	<0.001			
Center	1.42	1.01–2.01	0.047	0.743	1.14–2.49	0.009
South	1.57	1.18–2.09	0.002	1.269	1.58–2.99	<0.001
Islands	0.70	0.40–1.26	0.234			
Senior RD	5.51	3.95–7.69	<0.001	5.14	3.67–7.21	<0.001

OR, odds ratio; adjOR, adjusted odds ratio; CI, confidence interval; RD, resident doctor.

Regarding hospitalization, several regression models were applied to study associations. Analysis of discharge notes signed autonomously by RDs was carried out (Supplementary Table S4). The bivariable regression did not show any significant association, however, when adjusting in the multivariable model, seniority (adjOR 5.55, 95% CI 3.98–7.75, *p *< 0.001) and the South region (adjOR 1.61, 95% CI 1.20–2.16, *p *= 0.001) showed positive associations with the RD's autonomy in signing discharge notes.

In the same context, extra hours worked in the infirmary by RDs were analyzed recurring to logistic regressions and considering a cut off value of 72 h (Supplementary Table S5). The bivariable analysis showed negative associations with seniority (OR 0.75, 95% CI 0.58–0.69, *p *= 0.027) and the North region (OR 0.64, 95% CI 0.48–0.84, *p *= 0.001). Positive associations were found with the South region (OR 1.51, 95% CI 1.17–1.95, *p *= 0.001). The final multivariable regression confirmed the negative association with seniority (adjOR 0.74, 95% CI 0.57–0.96, *p *= 0.025).

Regarding 24 h-shifts in the Emergency Department, a logistic model was applied (Supplementary Table S6), with significant negative associations in the North (OR 0.53, 95% CI 0.42–0.67, *p *< 0.001) and Center region (OR 0.53, 95% CI 0.40–0.70, *p *< 0.001), while the South region (OR 2.48, 95% CI 2.01–3.06, *p *< 0.001) and seniority (OR 1.47, 95% CI 1.19–1.80, *p *< 0.001) were positively associated with working in these shifts. The multivariable model confirmed the negative associations in terms of region, namely in the North (adjOR 0.51, 95% CI 0.31–0.84, *p *= 0.008) and in the Center (adjOR 0.46, 95% CI 0.27–0.78, *p *= 0.004), and the positive association with seniority (adjOR 1.52, 95% CI 1.23–1.88, *p *< 0.001).

The logistic models were also applied to untaken days off after ER shifts in the weekend and holidays, with a cut off > 2 (Supplementary Table S7). The bivariable analysis showed positive associations with seniority (OR 1.38, 95% CI 1.09–1.75, *p *= 0.007) and the South region (OR 1.36, 95% CI 1.08–1.72, *p *= 0.010). These associations were confirmed in the multivariable analysis, namely seniority (adjOR 1.39, 95% CI 1.10–1.75, *p *= 0.007) and South region (adjOR 1.37, 95% CI 1.08–1.73, *p *= 0.010).

Another analysis was also performed in the primary healthcare context regarding the RD's performance of extra shifts for acute consultations after the regular opening hours of the primary healthcare center (complementary shifts), available in Supplementary Table S8. The initial analysis showed positive associations with the North region (OR 1.61, 95% CI 1.20–2.16, *p *= 0.002) and seniority (OR 2.91, 95% CI 2.15–3.04, *p *< 0.001), and negative association with the South (OR 0.67, 95% CI 0.50–0.91, *p *= 0.010). The final multivariable analysis confirmed that seniority (adjOR 2.97, 95% CI 2.19–4.03, *p *< 0.001) is most contributing factor, which increases the probability in performing these shifts.

Regarding studying time aside from regular working hours, RDs were asked how many hours they studied. For the regression, a cut off of 10 h was set (Supplementary Table S9), which returned negative associations with seniority (OR 0.83, 95% CI 0.69–0.99, *p *= 0.033) and the South region (OR 0.82, 95% CI 0.69–0.98, *p *= 0.030), also verified in the multivariable model for both variables—seniority (adjOR 0.83, 95% CI 0.69–0.98, *p *= 0.033) and South region (adjOR 0.82, 95% CI 0.69–0.98, *p *= 0.029).

Spendings with medical training were also evaluated. A cut off of EUR 1,500 was defined for the regression models (Table 6), with positive findings related to seniority (OR 1.24, 95% CI 1.04–1.48, *p *= 0.019) in the binary model, which were further confirmed in the multivariable model (adjOR 1.24, 95% CI 1.03–1.48, *p *= 0.021).

**Table 6:** Logistic regression model related to annual spending in training (cut off = EUR 1,500).

Variable	OR	95% CI	*p*-value	adjOR	95% CI	*p*-value
Region						
North	0.93	0.77–1.13	0.481			
Center	1.15	0.92–1.42	0.214			
South	0.93	0.78–1.12	0.436	1.15	0.93–1.43	0.209
Islands	1.25	0.81–1.92	0.318	1.31	0.85–2.02	0.229
Senior RD	1.24	1.04–1.48	0.019	1.24	1.03–1.48	0.021

OR, odds ratio; adjOR, adjusted odds ratio; CI, confidence interval; RD, resident doctor.

## Discussion

We highlight the fair representativeness of the sample, involving the participation of approximately 20% of all RDs in Portugal, with representation from all specialties.

### Working hours and unpaid and paid overtime

The medical residency involves a 40 h week schedule (1). However, the results from this study show that the actual working hours of RDs extend well beyond this period, and more than often this overtime is unpaid. RDs perform about 240 unpaid overtime hours annually, corresponding to a month and a half of unpaid extra work per year in the PNHS. Additionally, the vast majority of RDs work in the ED on weekends and holidays. In this case, they often give up the time off they are legally entitled to take to ensure the smooth running of the department to which they belong. This results in a median of two days of rest per month, corresponding to about 192 h per year. RDs who work in the ED perform an annual total of 432 unpaid overtime hours (240 overtime hours added to 24 untaken days off), which corresponds to more than two and a half months of unpaid overtime per year.

Regarding paid overtime, it should be noted that this corresponds to the smallest part of the total overtime worked by RDs, but even so this figure amounts to 144 annual overtime hours. Moreover, the working hours exceeding 40 h per week with no overtime pay was one of the reasons given by respondents to justify dropping out of medical residency, which raises further questions and the need of further investigation on the reasons to quit the programs.

When analyzing association between variables, seniority was associated with lower probability of doing more than fifteen hours of overtime unpaid work, whereas being a RD from the North or Center also revealed to be negatively associated with doing more than 23 h of overtime paid work. In the ER context, RDs from the South have a 1.38 higher probability of doing more than three hours of overtime unpaid work in shifts, whereas a 0.70 lower probability was found in the North and Center regions when considering 20 h of paid overtime work. In the infirmary, a negative association with seniority was found. These differences might be attributed to the unequal regional distribution of physicians, as described in the literature and national data (15, 16). However, these regional inequalities were previously described as littoral and countryside (15), and not according to geographic regions, which may indicate that more studies on human resources in health, especially on physicians, are needed. Nonetheless, as the rate of doctors per 100,000 inhabitants is higher in the North, the differences in overtime hours performed might be due to the level of shortage of doctors, which is higher in the Center and South when comparing to the other regions (16).

Another reason related to decreasing workload in overtime unpaid work among senior residents might be related to the hypothesis that the older the resident, the higher the probability of being scheduled to do paid overtime hours. While that might lower the available hours to perform unpaid overtime hours, it does not mean that senior residents work less—on the contrary, they might work more. This hypothesis requires further studies analyzing these specificities within different specialties.

Studies have been conducted in other countries that analyze working conditions for medical residents. Unpaid working hours are also reported among residents from the United States, which push them further into debt (17). In Poland, RDs reported high overtime, physical and mental strain at work as reasons not to pursue surgical specialties, highlighting the need to adjust the mode of education so that conditions are attractive for RDs (18). German RDs reported accepting positions with overtime hours over the ideal part-time positions (19), as acceptable work-life balance and remuneration and compensation for overtime are essential to maintain their satisfaction with the work environment (20).

The working environment proved to be essential for RDs. Overtime work in emergency shifts have been linked to increased levels of stress in RDs (21). However, capping working hours might not be enough to prevent burnout within this population, as other factors might also influence job quality and satisfaction (22). The current tendency among medical residents is to choose a specialty that allows a better work-life balance, with better working hours and fair pay, which has caused a shift in specialties selection among Japanese RDs (23).

### Autonomy in medical practice

In Portugal, residency programs require the direct mentoring of RD (1, 2). However, results demonstrate that during residency doctors have a significant degree of autonomy and responsibility, being an essential part of the functioning of a medical department.

Regarding the provision of consultations to patients, approximately 85% of the surveyed RDs provide consultations autonomously. Among the RDs who perform inpatient activities, almost all of them prescribe in-hospital medication (99.2%), prescribe outpatient medication (99%) and sign their own discharge summaries for patients (93.6%). The vast majority of RDs also perform invasive medical procedures (80.7%), including placement of central venous catheters, collection of biopsies and bone marrow aspirates, placement of temporary pacemakers, among others.

The vast majority of the surveyed RDs (87.1%) work in the ED. Regarding the workload, approximately 33% of them work 24 h emergency shifts (more than the 12 h stipulated in contract). There is also a high autonomy among RDs in these departments, as approximately 25% of the RDs cover emergency shifts without the presence of a specialist doctor. Furthermore, 75% of the RDs perform invasive medical procedures in the context of a medical emergency in the ED.

The inferential analysis on consultations showed a higher probability of doing them autonomously in senior RDs, in the Family Medicine specialty, and among those belonging to the Center and South regions. These differences might be related with the higher level of experience of senior RDs, as well as geographic inequalities of the rate of specialist Family Medicine doctors, which is lower in the Center, followed by the South (16).

Autonomy in signing discharge notes by RDs also found significant associations, with seniors having 5.55 times higher probability of doing so, and RDs working in the South region with a 1.61 higher probability. Seniority once again plays a significant role in the performance of autonomous tasks, and regional differences in the overall rates of doctors might again contribute to these findings (16).

Working in 24 h-ER shifts was also evaluated. Being a senior resident presented a positive association, whereas the North and Center regions were negatively associated with doing such shifts. Days off related to ER shifts also showed that senior RDs and those from the South had respectively a 1.39 and 1.37 higher probability of having more than two untaken days off per month, due to ER shifts performed on weekends or holidays. In the context of primary healthcare, complementary shifts also found a relation with seniority, with an adjusted OR of 2.97.

Seniority and regional inequalities explain these results, as the rate of doctors is highest in the North, and moderate in the Center region (16). The workload for RDs in EDs can also be explained by the medical demography of the PNHS (24). Not only do RDs represent about one third of all physicians in the PNHS, but they are also the youngest (25), thus increasing the proportion of RDs on emergency duty.

The Portuguese regulations also play a role in the overall function of the ER work. A medical specialist must be physically present in the ER doing clinical work, being uncommon to be on call. Due to this fact, the Portuguese legislation allows for doctors over 50 years of age to request exemption from working in emergency departments (26), which can further contribute to increasing the workload of RDs. As such, we believe that the role of the RD is vital for the functioning of the ED.

A German study reported that the main reason for decreased motivation within RDs were related to staff shortage and lack of support provided by supervisors and the administration (20), which are essential for training better healthcare professionals and avoiding their exodus from the public healthcare system into private practice.

### Training during residency

Regarding training in the medical residency, we found an almost total absence of hours dedicated to RD study during working hours (median of 0 h); the 40 h were used exclusively for care provision. RDs invest heavily in their training; 40.8% of them invest more than EUR 1,500 annually and 11.7% spend more than EUR 3,000 annually on their medical training. These amounts correspond to more than one or two months of work (respectively), considering the baseline net salaries of RDs (27). When assessing association with spending over EUR 1,500, seniority was found to have a 1.24 higher probability of spending over that amount.

RDs were also asked about the studying time after working hours. Negative associations were found between studying over ten hours with seniority and South region. This can be partially explained by the lower amount of time senior RDs have to dedicate to studying, adding to the fact that most residents in the South are also performing more overtime hours as concluded above. Studying time is considered as essential to train better healthcare professionals, and time dedicated for it is needed, raising concerns about the decrease of quality of future doctors. One strategy that might mitigate this is to allocate time within the residency for studying and developing research, which has proven to raise productivity among RDs (28).

Continuing education is valued by RDs, which is rather insufficient and of lower quality, as reported in this and other studies (20). There is a need to reduce individual costs with external training that burden RDs. Incorporating training opportunities within the residency can be suggested if they provide essential skills for the residency.

### Implications of findings

Our survey provided a portrait of the current working conditions in the Portuguese context, concerning overtime work, training opportunities, and autonomy in resident doctors. At the same time, some issues arise concerning labor legislation and Doctors' Union agreements, as overtime work is to be paid, 24 h ER shifts are not permitted, and compensatory resting period after ER shifts is mandatory (29, 30). Specifically, resident doctors are not allowed by the Portuguese law to perform autonomous work without supervision, even when they are in their last year of residency, contrary to what happens in other countries, such as the United Kingdom or the United States (31). While the Portuguese labor legislation can be protective of labor rights of healthcare professionals, due to the shortage of human resources, hospitals might be more permissive in regard to the fulfillment of the legislation and Doctors' union agreement, not penalizing residency and hospital directors for such non-compliance. All these issues must be addressed by the doctors themselves, as well as by Governmental bodies, Doctors' Unions and the Medical Council in order to ensure the well-being of residents and the safety of our patients.

On the other hand, most residents do not have a period assigned for training activities, including time allocated for presentations, development of scientific research, delivering training sessions. The only exception to this is the Family Medicine residency program, which in theory includes eight hours for such activities, however the increasing workload in primary healthcare centers might disregard it, as senior residents present lower periods of training activities. As such, there might be a need to understand whether such periods are significant to better outcomes in terms of residency evaluation and quality.

The Portuguese public administration sector provides fifteen paid days for training in case the employer deems it relevant. However, there are only a few courses that are considered as mandatory in the legislation, and these are required to be paid by the employing institution, as the resident doctor cannot successfully complete the residency program without approval in these courses. All the remaining courses are also considered training, such as presentations in conferences or papers published in scientific journals, which are paid by the resident. Note that all these extra activities have added value for the curriculum and to the final grade of the residency. It is of utmost importance to understand if these paid opportunities significantly impact the outcomes in the residency, so that some of these opportunities can be offered as a part of the residency, in order to reduce inequities among residents.

Other potential consequences of the working conditions analyzed in our sample of healthcare workers include the aspects related with dissatisfaction, loss of motivation and burn out. Working conditions can interfere with their personal life and their mental health, as reported in other studies (21–23). Symptoms of depression, anxiety and stress among residents might also have increased by the intense workload during the pandemic (32) and the subsequent pandemic fatigue. Moreover, around 9% of the burned-out doctors are more likely to have made at least one major medical error in the past three months, which impacts the quality of care and increases the burden on health systems (33).

Burnout also presents a severe cost to the economic sustainability of healthcare systems. In the US, the cost of physician turnover and reduced working hours due to burnout was estimated to be around 7,600 dollars per physician each year, summing up to 4.6 billion dollars yearly (34). This cost is likely to be higher in residents as this occurs earlier in the career, especially in European RDs, as the US presents a safer working environment, better career opportunities and more coping methods for emotional exhaustion, when compared to European countries (33). All these aspects highlight the need for a joint approach of all competent bodies in ensuring the protection of healthcare workers’ wellbeing, for the sake of the sustainability of health systems.

Additionally, a Portuguese study reported reasons for migration of doctors, with income and research opportunities as factors increasing migration intentions (35). As such, several countries across Europe need to invest in a systemic human resource planning in health and a reform in the respective health systems.

This paper aimed at providing a first glimpse of the workload carried out by medical residents in Portugal. This is particularly important due to the inexistence of a standardized methodology in place to evaluate the workload of doctors in Portugal, which is essential to evaluate performance and quality of care within the PNHS. Furthermore, such conclusions are useful to inform Doctors' Unions on ongoing issues regarding working hours and paid overtime hours, as well as quality of care, and instigate further investigation on such matters to improve doctors' working conditions in the PNHS.

The pandemic has changed the way healthcare professionals perceive and value their work. With the current human resources' crisis happening across Europe, with strikes and increasing short-staffed healthcare services (12), there is a need to comprehend several factors that contribute to this crisis, including income and working conditions of residents, as these are the future of the healthcare services.

### Limitations

The self-report format is the major limitation to this study, as it might lead to information bias, adding to the fact that the information collected pertained to September 2020, with a possibility of recall bias. However, the physicians were asked to base their answers on official records, which helped to mitigate these limitations. Selection bias might also be present as the survey was sent to RDs affiliated with the Independent Union of Portuguese Doctors and their representatives. Moreover, those who had their workload affected by the pandemic are more prone to fill in the form, which may also influence our results.

We were unable to account for the percentage of physicians who responded to the surveys and who are undergoing specialty training in the private sector. It is, however, widely accepted that it represents a residual percentage and that, in most cases, residency takes place in public services (36–38). Moreover, as the survey was applied during the pandemic, adherence might have been negatively impacted due to the increased workload.

Specialty might present as a possible confounding factor as it can influence the variables analyzed. However, adjusting for specialty was not always possible as there are over 45 specialties in Portugal and cluster analyses might not be significant in this context, as most RDs from all specialties were mobilized to work on COVID-19.

Finally, even though the selection and placement of RDs is carried out by the government, the medical residency can happen in both the public and private sector, with the majority happening in the public sector. This happens in an exclusivity regimen that can be voided for a period of time if requested by the employee. These factors were not accounted for in this research, as they were not the main aim of our study. However, differences in these contexts might influence working conditions and the quality of the residency program, which deserve further investigation. Furthermore, more studies on the economic impact of the residents' work in the health systems are needed, especially on medical errors and burnout.

## Conclusion

The results of this study showed the current state of RD's working conditions in Portugal. It is noteworthy that the great amount of time is dedicated to healthcare activities and do not include training time. RDs have a significant degree of autonomy in their clinical activity, and their personal and financial investment in their training is very high. We conclude that RDs play a vital role in the PNHS. Without their contribution, patient care would be permanently and severely compromised.

It is essential to invest in improving working conditions and the quality of post-graduate training of medical residents, namely to provide time and opportunities for RDs to dedicate to their education, which can be included in the residency program and not cause extra burden on RDs.

One must also highlight the need for better human resources planning within the public healthcare sector, with particular focus on doctors, allowing for a more balanced work schedule and avoiding increase of mental health issues among RDs, which further contributes to their dissatisfaction and loss of motivation, ultimately leading to their exit from the public health system.

## Data Availability

The original contributions presented in the study are included in the article/**Supplementary Material**, further inquiries can be directed to the corresponding author. Requests to access the datasets should be directed to: José Chen-Xu, josechenx@gmail.com.
