# Corrigendum: Medical residency in Portugal: a cross-sectional study on the working conditions

**DOI:** 10.3389/frhs.2024.1545815

**Published:** 2025-01-06

**Authors:** José Chen-Xu, Bruno Miranda Castilho, Bruno Moura Fernandes, Diana Silva Gonçalves, André Ferreira, Ana Catarina Gonçalves, Maycoll Ferreira Vieira, Andreia M. Silva, Fábio Borges, Mónica Paes Mamede

**Affiliations:** ^1^ Unidade de Saúde Pública, Agrupamento de Centros de Saúde Baixo Mondego, Coimbra, Portugal; ^2^ Comprehensive Health Research Centre, National School of Public Health, NOVA University of Lisbon, Lisbon, Portugal; ^3^ Cardiology Department, Hospital Distrital de Santarém, Santarém, Portugal; ^4^ Radiology Oncology Department, Centro Hospitalar Universitário de Coimbra, Coimbra, Portugal; ^5^ Unidade de Saúde Familiar ARS Médica, Agrupamento de Centros de Saúde Loures-Odivelas, Loures, Portugal; ^6^ Medical Oncology Department, Centro Hospitalar de Lisboa Ocidental, Lisboa, Portugal; ^7^ Infectious Diseases Department, Centro Hospitalar Universitário de Lisboa Central, Lisboa, Portugal; ^8^ Centros de Saúde de Santana e do Caniçal, Serviço de Saúde da Região Autónoma da Madeira, EPERAM, Madeira, Portugal; ^9^ General Surgery Department, Hospital da Horta, EPER, Açores, Portugal; ^10^ Unidade de Saúde Familiar S. Miguel-O-Anjo, Agrupamento de Centros de Saúde Ave-Famalicão, Famalicão, Portugal; ^11^ Anaesthesiology Department, Centro Hospitalar de Lisboa Central, Lisboa, Portugal

**Keywords:** medical education, residency, training, health policy, health economics, healthcare workers

**Corrigendum on**
Medical residency in Portugal: a cross-sectional study on the working conditions By Chen-Xu J, Miranda Castilho B, Moura Fernandes B, Silva Gonçalves D, Ferreira A, Gonçalves AC, Ferreira Vieira M, Silva AM, Borges F, Paes Mamede M (2023). Front. Health Serv. 3:1190357. doi: 10.3389/frhs.2023.1190357

**Error in Figure/Table**

In the published article, there was an error in Table 3 as published. The third line of the first column reads “1–500”, however it should read “1–1,000”. The corrected Table 3 and its caption appear below.

**Table 3:** Annual spending on training during residency.

Annual spending on training (EUR)	Number (percentage)
0	2 (0.1%)
1–1,000	795 (39.6%)
1,001–1,500	391 (19.5%)
1,501–2,000	294 (14.7%)
2,001–2,500	182 (9.1%)
2,501–3,000	107 (5.3%)
>3,000	235 (11.7%)

The authors apologize for this error and state that this does not change the scientific conclusions of the article in any way. The original article has been updated.

**Text Correction**

In the published article, there was an error. In the sub-section “Medical Training” from the Results section, there is a typo in the text.

A correction has been made to **Results**, *Medical Training*, Paragraph 2. This sentence previously stated:

“Most RDs (*n* = 795, 39.6%) reported spending up to EUR 500 per year in further training, followed by EUR 1,001–1,500 (*n* = 391, 19.5%) and EUR 1,501–2,000 (*n* = 294, 14.7%).”

The corrected sentence appears below:

“Most RDs (*n* = 795, 39.6%) reported spending up to EUR 1,000 per year in further training, followed by EUR 1,001–1,500 (*n* = 391, 19.5%) and EUR 1,501–2,000 (*n* = 294, 14.7%).”

The authors apologize for this error and state that this does not change the scientific conclusions of the article in any way. The original article has been updated.

