# Supplementary material for: Medical residency in Portugal: a cross-sectional study on the working conditions
Source: Front Health Serv. 2023 Dec 5;3:1190357. doi: 10.3389/frhs.2023.1190357 (PMC10728646; doi:10.3389/frhs.2023.1190357)
Supplement: Supplementary file 1 [file Table1.docx]

**Supplementary Material**

**S1 Table. Logistic Regression Model related to monthly paid hours (cut off = 23 hours) performed by RDs.**

| **Variable** | **OR** | **95% CI** | **p-value** | **adjOR** | **95% CI** | **p-value** |
| --- | --- | --- | --- | --- | --- | --- |
| Region  North  Center  South  Islands | 0.78  0.81  1.43  1.09 | 0.64 – 0.94  0.65 – 1.02  1.19– 1.71  0.70 - 1.68 | 0.011  0.068  <.001  0.707 | 0.70  0.70 | 0.57 – 0.85  0.55 – 0.89 | <0.001  0.003 |
| Senior RD | 1.09 | 0.91 – 1.31 | 0.338 |  |  |  |

**S2 Table. Logistic Regression Model related to unpaid overtime hours performed in the ER by residents (cut off = 3 hours).**

| **Variable** | **OR** | **95% CI** | **p-value** | **adjOR** | **95% CI** | **p-value** |
| --- | --- | --- | --- | --- | --- | --- |
| Region  North  Center  South  Islands | 0.82  0.82  1.38  0.84 | 0.61 – 1.08  0.57 – 1.16  1.06 – 1.79  0.42 – 1.69 | 0.161  0.263  0.017  0.620 | 1.03  1.376 | 0.71 – 1.50  1.06 – 1.79 | 0.877  0.017 |
| Senior RD | 1.03 | 0.79 – 1.35 | 0.810 |  |  |  |

**S3 Table. Logistic Regression Model related to paid overtime hours performed in the ER by residents (cut off = 20 hours).**

| **Variable** | **OR** | **95% CI** | **p-value** | **adjOR** | **95% CI** | **p-value** |
| --- | --- | --- | --- | --- | --- | --- |
| Region  North  Center  South  Islands | 0.54  1.09  1.43  2.51 | 0.43 – 0.69  0.83 – 1.45  1.15 – 1.78  1.37 – 4.61 | <0.001  0.530  0.001  0.003 | 0.57  2.10 | 0.45 – 0.72  1.14 – 3.88 | <0.001  0.018 |
| Senior RD | 1.34 | 1.08 – 1.67 | 0.008 | 1.34 | 1.07 – 1.67 | 0.010 |

**S4 Table. Logistic Regression Model related to discharge notes signed autonomously by RDs.**

| **Variable** | **OR** | **95% CI** | **p-value** | **adjOR** | **95% CI** | **p-value** |
| --- | --- | --- | --- | --- | --- | --- |
| Region  North  Center  South  Islands | 1.21  0.99  1.41  0.18 | 0.70 - 2.09  0.52 - 1.89  0.84 - 2.38  0.08 - 0.39 | 0.504  0.977  0.193  0.179 | 1.61 | 1.20 – 2.16 | 0.001 |
| Senior RD | 1.47 | 0.87 – 2.50 | 0.153 | 5.55 | 3.98 – 7.75 | <0.001 |

**S5 Table. Logistic Regression Model related to the monthly extra hours worked in the infirmary by RDs (cut off = 72 hours).**

| **Variable** | **OR** | **95% CI** | **p-value** | **adjOR** | **95% CI** | **p-value** |
| --- | --- | --- | --- | --- | --- | --- |
| Region  North  Center  South  Islands | 0.64  0.89  1.51  1.35 | 0.48 – 0.84  0.64 – 1.24  1.17 – 1.95  0.71 - 2.56 | 0.001  0.501  0.001  0.362 | 0.74  1.269 | 0.52 – 1.06  0.91 – 1.76 | 0.102  0.154 |
| Senior RD | 0.75 | 0.58 – 0.69 | 0.027 | 0.743 | 0.57 – 0.96 | 0.025 |

**S6 Table. Logistic Regression Model related to performance of 24 hour-shifts in the Emergency Department by RDs.**

| **Variable** | **OR** | **95% CI** | **p-value** | **adjOR** | **95% CI** | **p-value** |
| --- | --- | --- | --- | --- | --- | --- |
| Region  North  Center  South  Islands | 0.53  0.53  2.48  1.24 | 0.42 - 0.67  0.40 - 0.70  2.01 - 3.06  0.78 - 1.99 | <0.001  <0.001  <0.001  0.367 | 0.51  0.46  1.31 | 0.31 - 0.84  0.27 - 0.78  0.81 – 2.13 | 0.008  0.004  0.270 |
| Senior RD | 1.47 | 1.19 – 1.80 | <0.001 | 1.52 | 1.23 – 1.88 | <0.001 |

**S7 Table. Logistic Regression Model related to monthly untaken days off after ER shifts performed by RDs during weekends and holidays (cut off > 2).**

| **Variable** | **OR** | **95% CI** | **p-value** | **adjOR** | **95% CI** | **p-value** |
| --- | --- | --- | --- | --- | --- | --- |
| Region  North  Center  South  Islands | 1.23  0.86  1.36  0.86 | 0.95 – 1.58  0.64 – 1.16  1.08– 1.72  0.48 - 1.53 | 0.112  0.319  0.010  0.605 | 1.37 | 1.08 – 1.73 | 0.010 |
| Senior RD | 1.38 | 1.09 – 1.75 | 0.007 | 1.39 | 1.10 – 1.75 | 0.007 |

**S8 Table. Logistic Regression Model related to the performance of complementary shifts in the primary healthcare context.**

| **Variable** | **OR** | **95% CI** | **p-value** | **adjOR** | **95% CI** | **p-value** |
| --- | --- | --- | --- | --- | --- | --- |
| Region  North  Center  South  Islands | 1.61  1.09  0.67  0.47 | 1.20 - 2.16  0.79 - 1.50  0.50 - 0.91  0.22 - 1.00 | 0.002  0.609  0.010  0.050 | 1.40  0.75  0.49 | 0.96 - 2.03  0.51 - 1.09  0.22 - 1.11 | 0.077  0.132  0.087 |
| Senior RD | 2.91 | 2.15 – 3.04 | <0.001 | 2.97 | 2.19 – 4.03 | <0.001 |

**S9 Table. Logistic Regression Model related to the study hours aside from the regular working hours (cut off = 10 hours).**

| **Variable** | **OR** | **95% CI** | **p-value** | **adjOR** | **95% CI** | **p-value** |
| --- | --- | --- | --- | --- | --- | --- |
| Region  North  Center  South  Islands | 1.15  1.09  0.82  1.06 | 0.95 – 1.38  0.88 – 1.35  0.69 – 0.98  0.69 – 1.63 | 0.143  0.438  0.030  0.786 | 0.82 | 0.69 – 0.98 | 0.029 |
| Senior RD | 0.83 | 0.69 – 0.99 | 0.033 | 0.83 | 0.69 – 0.98 | 0.033 |
